# Huangqi-Danshen Decoction Ameliorates Adenine-Induced Chronic Kidney Disease by Modulating Mitochondrial Dynamics

**DOI:** 10.1155/2019/9574045

**Published:** 2019-01-01

**Authors:** Xinhui Liu, Shiying Huang, Fochang Wang, Lin Zheng, Jiandong Lu, Jianping Chen, Shunmin Li

**Affiliations:** ^1^Department of Nephrology, Shenzhen Traditional Chinese Medicine Hospital, Guangzhou University of Chinese Medicine, Shenzhen, Guangdong, China; ^2^Shenzhen Key Laboratory of Hospital Chinese Medicine Preparation, Shenzhen Traditional Chinese Medicine Hospital, Guangzhou University of Chinese Medicine, Shenzhen, Guangdong, China

## Abstract

Chronic kidney disease (CKD) is a leading public health problem with high morbidity and mortality. However, the therapies remain limited. Traditional Chinese medicine (TCM) has been used for treating kidney disease for thousands of years and is an effective alternative treatment for CKD patients in China and other Asian countries. In the present study, we aimed to investigate the effect and mechanism of Huangqi-Danshen decoction (HDD), a TCM herbal decoction, on treating CKD. CKD rat model was induced by adding 0.75% adenine to the diet for 4 weeks. HDD extract was administrated orally to CKD rats at the dose of 4.7 g/kg/d for consecutive 4 weeks in adenine-induced CKD rats. Kidney function was evaluated by the levels of serum creatinine (Scr) and blood urea nitrogen (BUN). The pathological changes of kidney tissues were observed by periodic acid-Schiff (PAS) and Masson's trichrome staining. The proteins expression of renal fibrosis and mitochondrial dynamics were determined and quantified by Western blot analysis. CKD rats showed obvious decline in renal function as evidenced by increased levels of Scr and BUN, which were blunted by HDD treatment. HDD could also improve tubular atrophy and interstitial fibrosis of CKD rats. Moreover, HDD downregulated fibronectin, type IV collagen, and *α*-smooth muscle actin expression in CKD rats. Furthermore, mitochondrial dynamics was disturbed in CKD rats, which manifested as increased mitochondrial fission and decreased mitochondrial fusion. HDD treatment restored mitochondrial dynamics in CKD rats by repressing dynamin-related protein 1 and Mid 49/51 expression, promoting mitofusin 2 expression, and suppressing optic atrophy 1 proteolysis. In conclusion, HDD could significantly retard CKD progression through modulating mitochondrial dynamics.

## 1. Introduction

Chronic kidney disease (CKD) is defined as abnormalities of kidney structure or function persisting for at least 3 months, regardless of the underlying cause [1]. The prevalence of all stages of CKD varies between 7 and 12% in the different regions of the world [1]. CKD contributes 1.35% of the global burden of disability life years lost, growing at a rate of 1% per year [2]. Despite this, there are relatively few options for the treatment of CKD. The mainstay therapeutic approach to retard progressive CKD or renal fibrosis is based on rennin-angiotensin system (RAS) blockade as well as blood pressure and glycemic control [3–5]. These interventions could not prevent the progression to end-stage renal disease (ESRD). In addition, the benefit of standard therapies varies across stages of CKD [6]. Therefore, mechanism investigation, discovering therapeutic targets, and searching effective medications are always necessary for CKD treatment.

Traditional Chinese medicine (TCM) is frequently used for treatment of CKD in China and many other Asian countries [7–10]. According to TCM theory, Qi deficiency and blood stasis (Qi-Xu-Xue-Yu) runs through the process of CKD development and progression. Therefore, replenishing Qi and activating blood (Yi-Qi-Huo-Xue) is the basic therapeutic principle of TCM in the treatment of CKD [11]. Huangqi-Danshen decoction (HDD) is composed of* Astragali Radix* (Huang-qi) served as replenishing Qi and* Salviae Miltiorrhizae* Radix et Rhizoma (Dan-shen) served as activating blood. Both Huang-qi and Dan-shen were firstly recorded in “Shennong Bencao Jing” (The Devine Farmer's Materia Medica, 300-200 B.C., Warring States Period to Han Dynasty) and were the most frequently prescribed herbs for treating CKD in clinical practise [12–14]. Pharmacological studies showed that Huang-qi has antioxidant, anti-inflammatory, immuneregulatory, anticancer, hypolipidemic, antihyperglycemic, hepatoprotective, expectorant, and diuretic effects [15]. Dan-shen has been proven to have various pharmacological activities, such as cardiovascular and cerebrovascular effects, antioxidative, neuroprotective, antifibrotic, antiinflammatory, and antineoplastic activities [16]. According to TCM theory, combined used of Huang-qi and Dan-shen will benefit to retard CKD progression by conjugating replenishing Qi and activating blood. In the present study, we tested this theory in adenine-induced CKD rat model and investigated underlying mechanism.

Mitochondria are the main energy-producing organelles in mammalian cells, but they also have a central role in deciding cell injury and death [17]. Since kidney is a high energy demanded organ and rich in mitochondria, mitochondrial dysfunction plays a critical role in the pathogenesis of kidney diseases [18]. Mitochondria are a class of dynamic organelles that constantly undergo fission and fusion [19]. Emerging evidence has demonstrated the alterations of mitochondrial dynamics in renal ischemia–reperfusion injury, nephrotoxicity, and hyperglycemia-induced kidney injury [20]. However, very little is known about the effect of mitochondrial dynamics on CKD. Hence, in the present study, we investigated the alterations of mitochondrial dynamics in a CKD model. Moreover, we studied how HDD could blunt CKD progression by modulating mitochondrial dynamics.

## 2. Materials and Methods

### 2.1. Preparation of HDD Water Extract

The herbal composition and proportion of HDD were summarized in Table 1. Raw herbs were purchased from Shenzhen Huahui Pharmaceutical Co., Ltd. (Shenzhen, China). The plant materials were authenticated by Jianping Chen based on their morphological characteristics.* Astragali Radix* (30 g) and* Salviae Miltiorrhizae* Radix et Rhizoma (15 g) were weighed and boiled twice in 8 times of ddH2O (w/v) for 1 hour per time. The HPLC profile of HDD extract was provided in Supplementary Figure 1. For the convenience of administration, the extract was dried by freeze dryer and stored at -80°C. Before the treatment, the freeze-dried powder was redissolved with ddH_2_O to get HDD extract.

### 2.2. Animals and Experimental Treatment

All animal experiments were conducted with protocols approved by the Ethics Committee of Shenzhen Traditional Chinese Medicine Hospital, Guangzhou University of Chinese Medicine. Twenty-four male Spraque-Dawley (SD) rats weighting 180-220g were purchased from Guangdong Medical Laboratory Animal Center (Foshan, China, permission no. SCXK (YUE) 2013-0002). The rats were maintained at a constant temperature (22°C–25°C) and humidity (40%–70%) with a 12-hour light/12-hour dark cycle. After one week of acclimatization, all rats were randomly divided into three groups: (1) control group (n=8), (2) CKD group (n=8), and (3) HDD-treated group with CKD (n=8). CKD was induced in rats by feeding adenine (Sigma-Aldrich, St Louis, MO, USA) in feed at a concentration of 0.75% w/w for 4 weeks [21]. Treatment group rats received 4.7 g/kg/d dose of HDD extract orally for 4 weeks with simultaneous adenine feeding. Control group rats received normal adenine-free feed for 4 weeks. During the experiment, 2 rats died in the CKD group, but no rats died in other groups. At the end of study, all rats were anesthetized, and blood samples were obtained by cardiac puncture. The rats were euthanized by cervical dislocation without regaining consciousness. Kidneys were removed and preserved for histological analysis and Western blot analysis.

### 2.3. Biochemical Analysis

Serum creatinine (Scr) and blood urea nitrogen (BUN) were measured by creatinine serum detection kit and BUN detection kit (StressMarq Biosciences, British Columbia, Canada), respectively, following the manufacturer's instructions.

### 2.4. Histological Examination

Rat kidney tissue was fixed with 4% buffered paraformaldehyde (pH 7.4) at 4°C overnight, dehydrated in graded alcohols, and embedded in paraffin. The paraffin-embedded kidneys were cut into 2 mm sections and stained with periodic acid-Schiff (PAS) and Masson's trichrome stains for the evaluation of pathological changes. The tubular atrophy score in PAS staining was performed as previously described [22]. Quantitative analysis of fibrotic area was conducted by Image J software (NIH, Bethesda, MD, USA). Tubular atrophy score and fibrotic area measurement were performed on at least 10 fields (200×) in each slide and six rats in each group by a colleague who was blinded to the study design.

### 2.5. Western Blot Analysis

The kidney cortexes were homogenized in lysis buffer and measured protein concentration as our previously described [23]. Equal amounts of kidney cortex lysates were loaded and electrophoresed through 7% or 10% SDS-PAGE gels and were then transferred to nitrocellulose membranes or polyvinylidene difluoride membranes (Millipore, Billerica, MA, USA). After being blocked in 5% nonfat milk for 1 hour at room temperature, the membranes were incubated with primary antibodies at 4°C overnight. Then, the membranes were incubated in horseradish peroxidase (HRP)-conjugated anti-mouse IgG or HRP-conjugated anti-rabbit IgG (Life Technologies, Carlsbad, CA, USA) for 1 hour at room temperature. HRP activity was visualized using Clarity Western ECL Substrate and a ChemiDoc MP Imaging System (Bio-Rad Laboratories, Hercules, CA, USA). Image Lab software version 5.1 was used for densitometric analysis (Bio-Rad Laboratories, Hercules, CA, USA). The primary antibodies used in this study included dynamin-related protein 1 (Drp-1, 1:1000), mitofusin 2 (Mfn-2, 1:1000), *α*-tubulin (1:1000) (Cell Signaling Technology, Beverly, MA, USA), optic atrophy 1 (OPA-1, 1:2000) (BD Biosciences, San Jose, CA, USA), fibronectin (FN, 1:250), type IV collagen (Col-IV, 1:250) (abcam, Cambridge, MA, USA), *α*-smooth muscle actin (*α*-SMA, 1:1000) (Sigma-Aldrich, St Louis, MO, USA), glyceraldehyde-3-phosphate dehydrogenase (GAPDH, 1:5000), Mid 49 (1:500), and Mid 51 (1:500) (Proteintech, Wuhan, China).

### 2.6. Immunofluorescence Analysis

The paraffin-embedded kidneys were cut into 6 *μ*m sections, dewaxed, and rehydrated. After antigen retrieval in 10 mM sodium citrate (pH 6.0), the sections were incubated with block buffer (5% BSA in PBS) for 1 hour at room temperature. Then, the sections were stained with anti-Drp-1 (1:50) and anti-TOM 20 (1:100) at 4°C overnight followed by appropriate secondary antibodies. Nuclei were counterstained with the fluorescent dye 4′,6-diamidino-2-phenylindole (DAPI). In all cases, antibody negative controls were used to ensure the truth of positive staining. All images were captured by fluorescence microscope (Nikon, Japan).

### 2.7. Statistical Analysis

All data are presented as mean ± SEM. The statistical differences among groups were analyzed by one-way ANOVA followed by post hoc analysis with the Least Significant Difference (LSD) test or the Games-Howell test. The value of P<0.05 was considered statistically significant. Data were analyzed using SPSS 16.0 statistics software (SPSS Inc., Chicago, IL, USA).

## 3. Results

### 3.1. HDD Improved Renal Function in CKD Rats

**Figure 1:**
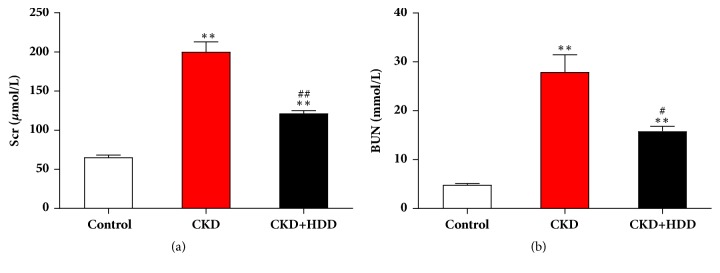
HDD improved renal function in CKD rats. The levels of Scr (a) and BUN (b) in different groups. Data are presented as the means ± SEM, n=6 rats per group (^*∗∗*^*P* < 0.01 compared with the control group; ^#^*P* < 0.05, ^##^*P* < 0.01 compared with the CKD group).

Renal function was assessed by Scr and BUN levels. Compared with the control group, Scr and BUN levels in the CKD group were significantly elevated (*P*<0.01). Administration of HDD obviously reduced Scr (*P*<0.01) and BUN (*P*<0.05) levels in CKD rats (Figure 1). Moreover, HDD had no effect on liver function as estimated by the levels of aspartate transaminase (AST) and alanine transaminase (ALT), which means the treatment dosage of HDD in current study is safe (Supplementary Table 1). These data indicated that CKD model was established successfully, and HDD prevented renal function decline in CKD rats.

### 3.2. HDD Ameliorated Renal Pathological Injury in CKD Rats

**Figure 2:**
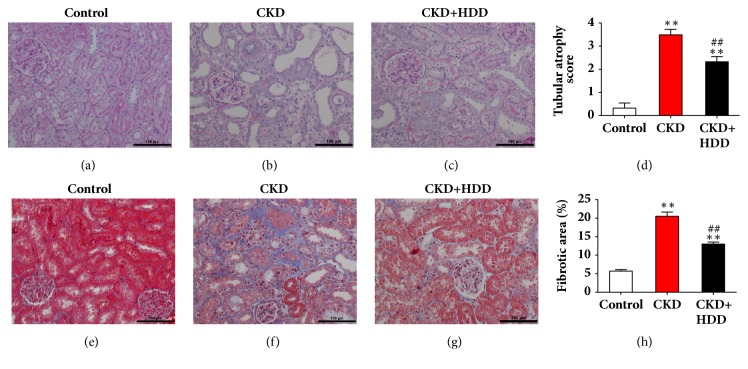
HDD ameliorated renal pathological injury in CKD rats. (a) Normal kidney structure in PAS staining of the control group. (b) Obvious tubular atrophy in the CKD group. (c) Reduced tubular atrophy in the CKD+HDD group. (d) Tubular atrophy score. (e) Normal kidney structure in Masson staining of the control group. (f) Massive collagen deposition in the CKD group. (g) Reduced collagen deposition in the CKD+HDD group. (h) Quantitative analysis of fibrotic area. All images are shown at identical magnification, ×200, scale bar=100*μ*m. Data are presented as the means ± SEM, n=6 rats per group (^*∗∗*^*P* < 0.01 compared with the control group; ^##^*P* < 0.01 compared with the CKD group).

The renal pathological injury in CKD is characterized by tubular atrophy and interstitial fibrosis. In PAS staining, CKD rats showed massive tubular atrophy, which could be improved by HDD treatment (Figures 2(a)–2(d)). Masson staining displayed obvious interstitial fibrosis in the CKD group, which was almost 4 times of the control group in quantitative analyses (*P*<0.01). HDD treatment reduced collagen deposition in Masson staining by 36% (*P*<0.01) (Figures 2(e)–2(h)). These data indicated that HDD protected kidney structure in CKD rats.

### 3.3. HDD Inhibited Renal Fibrosis in CKD Rats

**Figure 3:**
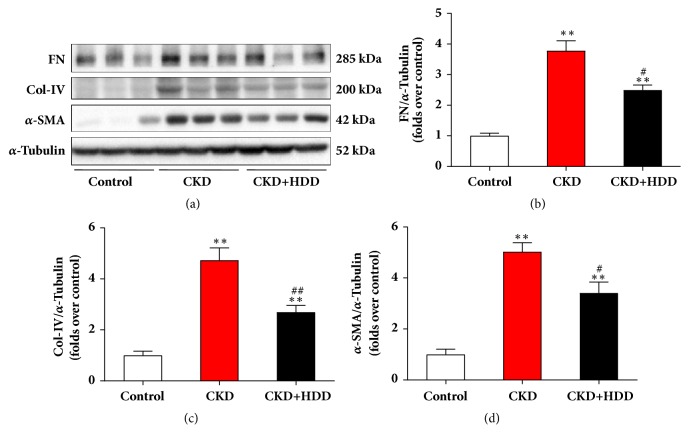
HDD inhibited fibrotic markers expression in CKD rats. (a) Representative Western blot images of FN, Col-IV, and *α*-SMA. (b-d) Densitometric analysis of FN, Col-IV, and *α*-SMA protein expression, respectively, normalized to *α*-Tubulin content. Data are presented as the means ± SEM, n=6 rats per group (^*∗∗*^*P* < 0.01 compared with the control group; ^#^*P* < 0.05, ^##^*P* < 0.01 compared with the CKD group).

Renal fibrosis is the common and final pathway from CKD to ESRD. We further explored the effect of HDD on fibrosis by evaluation of fibrotic markers expression. As shown in Figure 3, the expression of FN, Col-IV, and *α*-SMA was all upregulated in the CKD group (*P*<0.01). In contrast, HDD treatment significantly restored these proteins expression. These data provided further evidence of the beneficial effect of HDD on kidney structure.

### 3.4. HDD Modulated Mitochondrial Dynamics in CKD Rats

**Figure 4:**
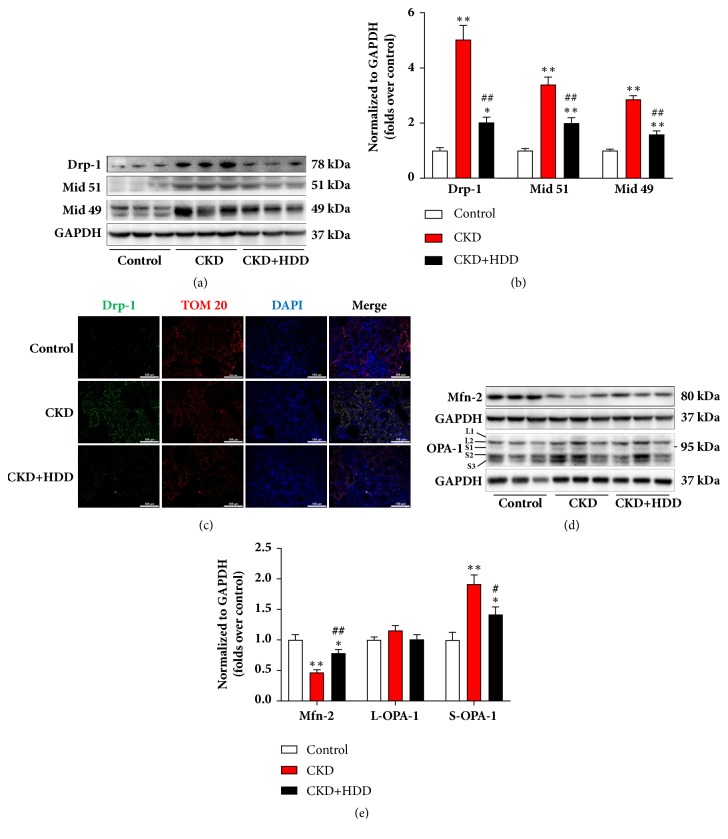
HDD decreased mitochondrial fission and increased mitochondrial fusion in CKD rats. (a) Representative Western blot images of Drp-1, Mid 51, and Mid 49. (b) Densitometric analysis of Drp-1, Mid 51, and Mid 49 protein expression, normalized to GAPDH content. (c) Representative immunofluorescence images indicating the colocalization of Drp-1 and TOM 20. Green corresponds to Drp-1, red corresponds to TOM 20, and blue corresponds to nuclear staining. All images are shown at identical magnification, ×200, scale bar=100 *μ*m. (d) Representative Western blot images of Mfn-2 and OPA-1 spectrum. (e) Densitometric analysis of Mfn-2, L-OPA-1, and S-OPA-1 protein expression, normalized to GAPDH content. Data are presented as the means ± SEM, n=6 rats per group (^*∗*^*P* < 0.05, ^*∗∗*^*P* < 0.01 compared with the control group; ^#^*P* < 0.05, ^##^*P* < 0.01 compared with the CKD group).

The balance of mitochondrial fission and fusion maintains mitochondrial homeostasis and function. We further investigated the status of mitochondrial dynamics in CKD and the regulating effect of HDD. Western blotting revealed that the expression levels of Drp-1, Mid 51, and Mid 49, the main regulators of mitochondrial fission, were significantly upregulated in the CKD group (Figures 4(a) and 4(b)). Immunofluorescence analysis showed that more Drp-1 colocalized with TOM 20, a mitochondrial marker, in the CKD group, which indicated more Drp-1 translocated to mitochondria to perform fission in CKD rats (Figure 4(c)). HDD administration restored the protein abundance of Drp-1, Mid 51, and Mid 49 and reduced translocation of Drp-1 to mitochondria (Figures 4(a)–4(c)). We next examined the expression of core components for mitochondrial fusion. The results showed that Mfn-2 was downregulated in the CKD group and partially restored by HDD treatment (Figures 4(d) and 4(e)). Two long isoforms (L1 and L2) and three short isoforms (S1, S2, and S3) of OPA-1 were detected in our study (Figure 4(d)). There was no significant difference in the expression of long isoforms of OPA-1 (L-OPA-1) among groups. But, there was a marked accumulation of short isoforms of OPA-1 (S-OPA-1) in the CKD group, indicative of OPA-1 proteolysis. Notably, the OPA-1 proteolysis was suppressed in the CKD+HDD group (Figures 4(d) and 4(e)). Excessive proteolysis leads to the inactivation of OPA-1 followed by the arrest of inner membrane fusion, contributing to inner membrane cleavage [24]. Therefore, these data collectively indicated that renal mitochondria were more prone to fission rather than fusion in our CKD model and HDD could improve this balance.

## 4. Discussion

Adenine-induced CKD rat model has been widely used for revealing the action mechanism of CKD and evaluating efficiency of TCM on CKD [25–30]. In the present study, HDD can improve renal function and attenuate renal pathological injury in the adenine-induced CKD rats. Moreover, HDD was found to modulate mitochondrial dynamics in the adenine-induced CKD rats manifested as inhibition of mitochondrial fission and promotion of mitochondrial fusion.

Mitochondria are highly dynamic organelles undergoing coordinated cycles of fission and fusion, referred as ‘mitochondrial dynamics', in order to maintain their shape, function, inheritance and quality control [19]. Defects in mitochondrial dynamics have been associated with many biological processes such as apoptosis, autophagy, metabolism, development, and aging [31]. Mitochondrial dynamics in mammalian is governed by fission mediators (such as Drp-1, Mid 49/51, and mitochondrial fission factor) and fusion proteins (such as Mfn-1/2 and OPA-1) [32]. Emerging evidence suggested a pathogenic role of mitochondrial dynamics in renal diseases. Gall et al. investigated conditionally deleted MFN2 gene in the kidney of mice and isolated proximal tubular cells. They found these cells showed obvious mitochondrial fragmentation and were sensitive to mitochondrial outer membrane injury and apoptosis following metabolic stress by adenosine triphosphate depletion [33]. Currently, studies on the effect of mitochondrial dynamics on CKD mainly focus on diabetic nephropathy (DN), a leading cause of ESRD. Sun et al. demonstrated a markedly mitochondrial fragmentation along with cristae remodeling during tubular cell apoptosis in diabetic mouse kidneys and high glucose induced HK-2 cells [34]. Another study also found that hyperglycemia-induced mitochondrial fission by promoting Drp-1 recruitment to the mitochondria in DN mouse model [35]. Recently, Zhan et al. demonstrated that mitochondrial fragmentation was an important pathogenic feature of tubular cell injury in human DN [36]. In line with previous studies, our data demonstrated an alteration of mitochondrial dynamics shown as increased fission and decreased fusion in adenine-induced CKD rats. However, in an early 5/6 nephrectomized CKD model, Aparicio-Trejo et al. found that 5/6 nephrectomy shifted mitochondrial dynamics to fusion [37]. Apart from model-specific differences, the duration of CKD (4 weeks versus 24 hours) maybe also associated with these distinct results.

Mounting evidence has demonstrated that TCM has been widely applied in clinic and established as an effective therapy for the treatment of CKD [38–42]. HDD, a Chinese herbal decoction, was established according to the TCM theory of replenishing Qi and activating blood, which is the basic therapeutic principle of TCM in the treatment of CKD. Since* Astragali Radix* and* Salviae Miltiorrhizae* Radix are representative herbs of Qi-regulating and blood-regulating respectively, our findings provide further evidence in CKD rats for previous patient-based study. The biological effects of* Astragali Radix* have been investigated in several animal models of kidney disease with the effects of anti-inflammation [43] and immune regulation [44].* Salviae Miltiorrhizae* Radix and its active components could also attenuate kidney injury in streptozotocin-induced diabetic rats [45] and 5/6 nephrectomized rats [46]. Moreover, several studies have demonstrated salvianolic acid A exhibited a beneficial protective effect on glomerulus damage and renal fibrosis [47, 48]. Our study confirmed the renoprotective effect of* Astragali Radix* and* Salviae Miltiorrhizae* Radix by combining application of these two herbs as HDD. Furthermore, our data showed that HDD restored the balance between mitochondrial fission and fusion in CKD rats. Posttranslational modifications of the core components are the main regulatory mechanisms of mitochondrial dynamics, including phosphorylation, ubiquitination, sumoylation, S-nitrosylation, and proteolysis [49]. It is postulated that HDD regulates mitochondrial dynamics by posttranscriptional modification of related proteins. However, the putative target and pathway of HDD need further detailed mechanistic studies.

## 5. Conclusions

In conclusion, HDD could significantly retard CKD progression, which might be associated with modulation of mitochondrial dynamics.

## Figures and Tables

**Table 1:** The herbal composition and proportion of HDD.

Botanical name	Herbal name	Chinese name	Voucher number	Dosage
*Astragalus membranaceus *(Fisch.) Bge. var. *mongholicus* (Bge.) Hsiao	*Astragali Radix*	Huang-Qi	2010015Z	30 g
*Salvia miltiorrhiza *Bunge	*Salviae Miltiorrhizae* Radix et Rhizoma	Dan-Shen	2010006Z	15 g

## Data Availability

The data used to support the findings of this study are available from the corresponding author upon request.

## Supplementary Materials

Supplementary Materials
** Supplementary Figure** **1**. Typical LC-MS chromatogram of HDD. (a) Structures of chemical markers analyzed in HDD extract, including calycosin 7-O-*β*-glucoside (1), salvianolic acid B (2), astragaloside IV (3), protocatechualdehyde (4), caffeic acid (5), rosmarinic acid (6), and lithospermic acid (7). (b) The representative LC-MS chromatograms of mixed standards and HDD extract.** Supplementary Table****1**. The levels of aspartate transaminase (AST) and alanine transaminase (ALT) in different groups.
