# Supplementary material for: Huangqi-Danshen Decoction Ameliorates Adenine-Induced Chronic Kidney Disease by Modulating Mitochondrial Dynamics
Source: Evid Based Complement Alternat Med. 2019 Jan 1;2019:9574045. doi: 10.1155/2019/9574045 (PMC6332985; doi:10.1155/2019/9574045)
Supplement: Supplementary Materials — Supplementary Figure 1. Typical LC-MS chromatogram of HDD. (a) Structures of chemical markers analyzed in HDD extract, including calycosin 7-O-β-glucoside (1), salvianolic acid B (2), astragaloside IV (3), protocatechualdehyde (4), caffeic acid (5), rosmarinic acid (6), and lithospermic acid (7). (b) The representative LC-MS chromatograms of mixed standards and HDD extract. Supplementary Table1. The levels of aspartate transaminase (AST) and alanine transaminase (ALT) in different groups. [file 9574045.f1.pdf]

Supplementary Figure 1. The HPLC profile of HDD extract

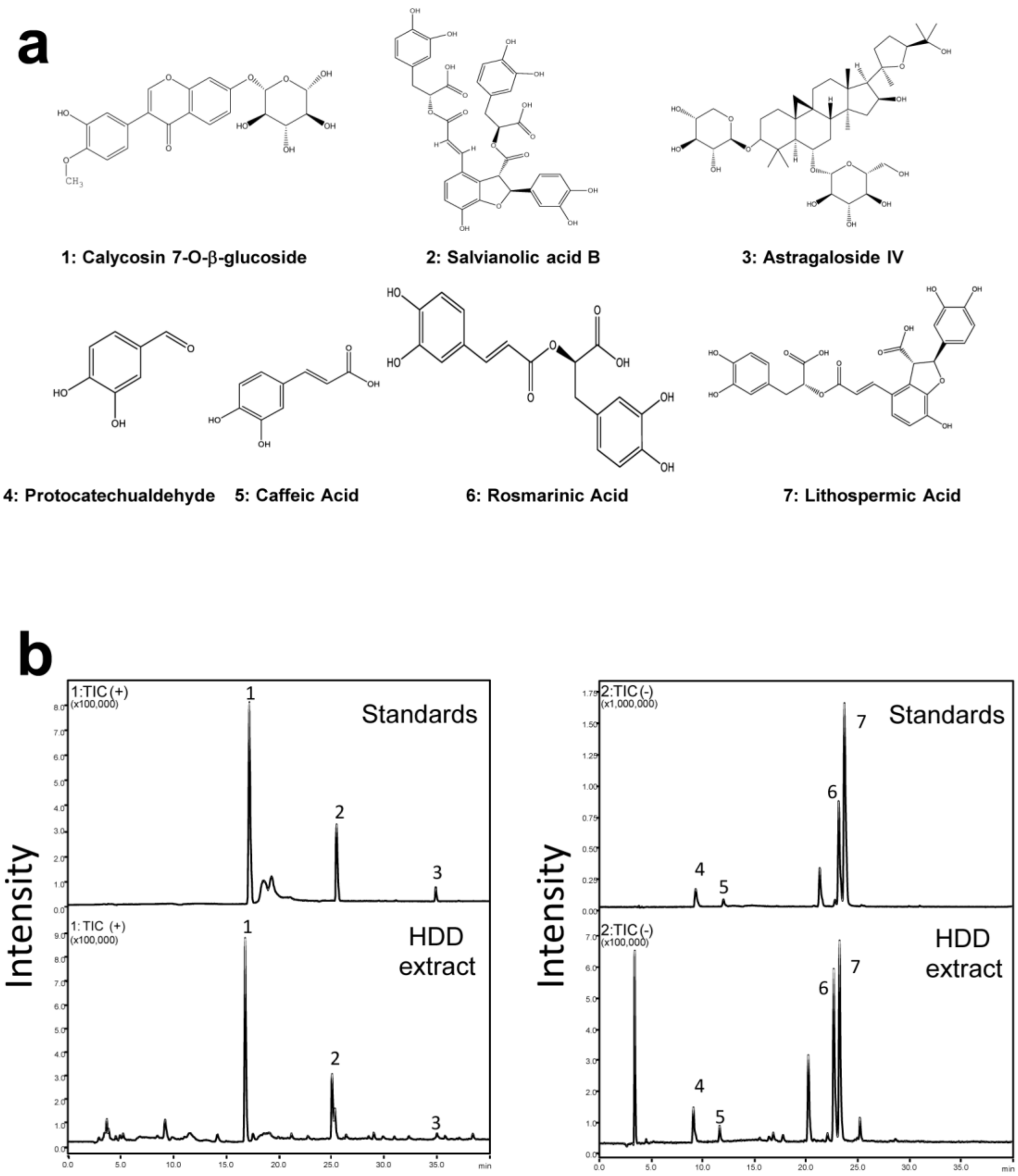

Supplementary Table 1. Liver function data

Group	AST (U/L)	ALT (U/L)
Control	91.3 ±6.7	28.6 ±1.0
CKD	90.8 ±3.3	28.1 ±1.6
CKD+HDD	91.6 ±4.6	27.4 ±0.6

Data are presented as the means ± SEM, n=6 rats per group.
